# Exploring the Thioredoxin System as a Therapeutic Target in Cancer: Mechanisms and Implications

**DOI:** 10.3390/antiox13091078

**Published:** 2024-09-04

**Authors:** Rebecca Seitz, Deniz Tümen, Claudia Kunst, Phillip Heumann, Stephan Schmid, Arne Kandulski, Martina Müller, Karsten Gülow

**Affiliations:** Department of Internal Medicine I, Gastroenterology, Hepatology, Endocrinology, Rheumatology, Immunology, and Infectious Diseases, University Hospital Regensburg, 93053 Regensburg, Germany; rebecca.seitz@stud.uni-regensburg.de (R.S.); deniz.tuemen@klinik.uni-regensburg.de (D.T.); claudia.kunst@ukr.de (C.K.); phillip.heumann@ukr.de (P.H.); stephan.schmid@ukr.de (S.S.); arne.kandulski@ukr.de (A.K.); martina.mueller-schilling@ukr.de (M.M.)

**Keywords:** thioredoxin (Trx), thioredoxin reductase (TrxR), reactive oxygen species (ROS), oxidative stress, anti-oxidative defence, cancer therapy

## Abstract

Cells constantly face the challenge of managing oxidants. In aerobic organisms, oxygen (O_2_) is used for energy production, generating reactive oxygen species (ROS) as byproducts of enzymatic reactions. To protect against oxidative damage, cells possess an intricate system of redox scavengers and antioxidant enzymes, collectively forming the antioxidant defense system. This system maintains the redox equilibrium and enables the generation of localized oxidative signals that regulate essential cellular functions. One key component of this defense is the thioredoxin (Trx) system, which includes Trx, thioredoxin reductase (TrxR), and NADPH. The Trx system reverses oxidation of macromolecules and indirectly neutralizes ROS via peroxiredoxin (Prx). This dual function protects cells from damage accumulation and supports physiological cell signaling. However, the Trx system also shields tumors from oxidative damage, aiding their survival. Due to elevated ROS levels from their metabolism, tumors often rely on the Trx system. In addition, the Trx system regulates critical pathways such as proliferation and neoangiogenesis, which tumors exploit to enhance growth and optimize nutrient and oxygen supply. Consequently, the Trx system is a potential target for cancer therapy. The challenge lies in selectively targeting malignant cells without disrupting the redox equilibrium in healthy cells. The aim of this review article is threefold: first, to elucidate the function of the Trx system; second, to discuss the Trx system as a potential target for cancer therapies; and third, to present the possibilities for inhibiting key components of the Trx system, along with an overview of the latest clinical studies on these inhibitors.

## 1. Introduction

Oxygen (O_2_) became crucial for life on Earth with the emergence of photosynthesis by archaebacteria. To utilize oxygen for energy production, organisms had to develop protective mechanisms against oxidation by O_2_ and reactive oxygen species (ROS) [1,2,3,4].

All cells have to balance the generation of ROS and their elimination by cellular antioxidant networks. The primary source of ROS is mitochondrial metabolism. Cancer cells have higher ROS levels than healthy cells due to uncontrolled proliferation and high metabolic rates. Elevated levels of ROS render cells vulnerable to oxidative stress. This weakness could be exploited to target and combat malignant cells. Therefore, there is growing interest in modulating cellular redox equilibrium as a potential strategy for cancer treatment [5,6,7,8].

The primary cellular ROS include superoxide anions (O_2_^•−^), hydroxyl radicals (^•^OH), and hydrogen peroxide (H_2_O_2_) [3,4,9,10,11]. In general, ROS production begins with electron transfer to O_2_, forming O_2_^•−^. O_2_^•−^ is highly reactive, with a short half-life of about 1 μs and limited diffusion through membranes due to its charge [3,4]. In aqueous environments, O_2_^•−^ rapidly converts to H_2_O_2_. Although not a radical, H_2_O_2_ is classified as an ROS. Compared to other ROS, it has a longer half-life of about 1 ms and can diffuse through cell membranes, and it is rather specific, oxidizing primarily free thiols [3,4]. H_2_O_2_ can be converted into water (H_2_O). This occurs via enzymes such as catalase, which transforms H_2_O_2_ into O_2_ and H_2_O [9,12,13]. Another key factor in H_2_O_2_ removal is the tripeptide glutathione, which acts as an effective oxidative scavenger in its reduced form (GSH) [9,12,14]. In addition to GSH, thioredoxin (Trx) acts as an essential disulfide reductase, reversing thiol oxidation. Trx is an important component of the cellular redox system, playing a crucial role in regulating various redox signaling pathways. It is also increasingly recognized as an important modulator of tumor development and tumor growth [14,15,16,17].

This review aims to provide a comprehensive understanding of the role of the Trx system in cellular processes. The review will highlight how Trx contributes to cellular redox homeostasis and its broader implications in health and disease by discussing its function. The second goal of the review is to explore the potential of the Trx system as a therapeutic target in cancer treatment. The final objective is to present different strategies for inhibiting key components of the Trx system and to provide an overview of the latest clinical studies involving these inhibitors.

## 2. The Thioredoxin System

The thioredoxin system, comprising NADPH, Trx, and thioredoxin reductase (TrxR), is highly conserved in all eukaryotic cells [18]. It plays a crucial role in regulating various cellular functions, including the activity of transcription factors, DNA synthesis (as a hydrogen donor for ribonucleotide reductase (RNR)), and cell growth through the activation of kinase cascades [19,20]. 

### 2.1. Thioredoxin

**Figure 1:**
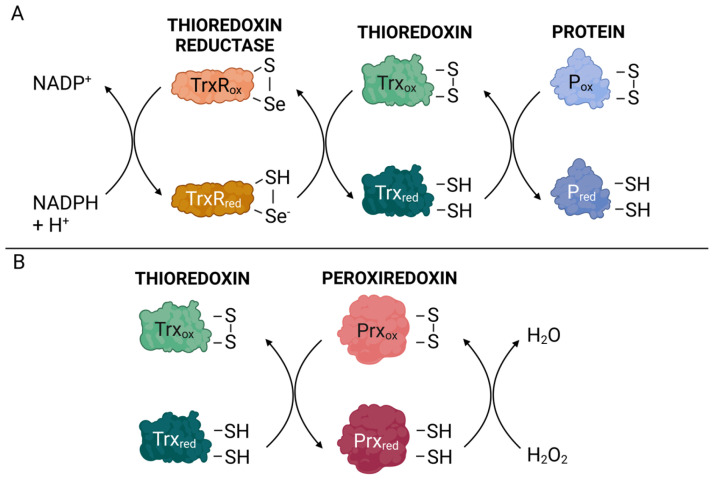
Schematic representation of the thioredoxin (Trx) redox system. (**A**) Reduced Trx catalyzes the reduction of disulfide bonds (S-S) in oxidized proteins. In this process, Trx is oxidized and subsequently reduced by thioredoxin reductase (TrxR). (**B**) Peroxiredoxin (Prx) converts H_2_O_2_ directly into water, becoming oxidized at a cysteine residue. The reduced Redox state of Prx is restored by reduction by Trx. red = reduced; ox = oxidized. The figure was created with the assistance of BioRender.com.

Trx is a small protein of 12 kDa, composed of five β-strands surrounded by four α-helices. This structure is conserved across the oxidoreductase superfamily [18]. In mammalian cells, three Trx isoforms have been identified: cytosolic Trx (Trx1), mitochondrial Trx (Trx2), and spermatozoa Trx (SpTrx). The redox-active site (CGPC) is conserved in all Trx proteins, typically located between the β2-strand and the N-terminal portion of α2 [21]. Human Trx1 has five cysteine (Cys) residues: Cys32 and Cys35 in the active center of the protein and Cys62, Cys67, and Cys73 outside the active center [18,21]. Cys73 regulates the overall activity of Trx [22,23,24,25,26]. The active form of Trx is the reduced dithiol form (Trx-(SH)_2_). Trx-(SH)_2_ can interact with target proteins and reduce disulfide bonds (-S-S-) within these proteins. During this process, the free thiols at the active site of Trx are oxidized, forming a disulfide bond between Cys32 and Cys35 (Trx-S2). This disulfide bond is then reduced back to the dithiol form by TrxR using NADPH as a co-factor, thus restoring the activity of Trx [18,19,27] (Figure 1A). In addition to reducing disulfide bonds in target proteins, Trx directly influences redox balances. Trx can transfer electrons to peroxiredoxins (Prx) to remove H_2_O_2_, ROOH, and ONOO^−^ and thus eliminate oxidative stress [27,28,29] (Figure 1B). Furthermore, Trx serves as the hydrogen donor for methionine sulfoxide reductases. These enzymes are responsible for reducing oxidized methionine, thereby repairing damage caused by oxidation [30,31,32,33].

In addition to Trx, there is also the thioredoxin-related protein of 14 kDa (Trp14), which should also be briefly mentioned here. Trp14 is a highly conserved oxidoreductase that is ubiquitously expressed [34,35]. It serves as a substrate for TrxR1, which can reduce Trp14 and restore its activity [35]. Unlike Trx, Trp14 does not influence the activities of ribonucleotide reductase, peroxiredoxins, or methionine sulfoxide reductases. Trp14 reduces L-cystine and thereby directly affects glutathione synthesis, which in turn impacts the cellular redox equilibrium [34].

### 2.2. Thioredoxin Reductase

**Figure 2:**
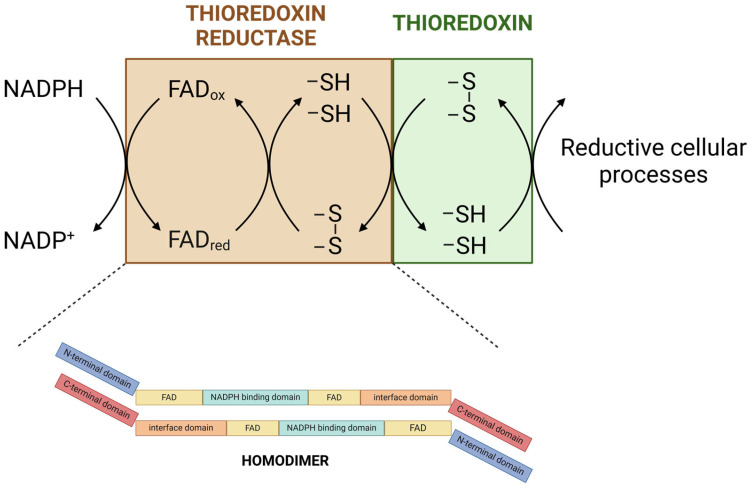
Schematic representation of the reaction catalyzed by the thioredoxin reductase homodimer. Reduction equivalents from NADPH are transferred to the flavin cofactor, then to the disulfide Cys135-Cys138 of thioredoxin reductase TrxR, and finally to the disulfide Cys32-Cys35 of thioredoxin (Trx). The figure was created with the assistance of BioRender.com.

TrxRs are essential for the Trx system, catalyzing the reduction of oxidized Trx to its active form using electrons derived from NADPH (Figure 1A). TrxR homodimeric proteins belong to the flavoprotein family of pyridine nucleotide disulfide oxidoreductases, which contain a prosthetic FAD group, an NADPH-binding site, and a dithiol/disulfide redox-active site. The active site of mammalian TrxR contains a selenocysteine residue, which is essential for efficient catalysis and distinguishes it from other oxidoreductases [17,27] (Figure 2).

In humans, TrxR exists in three isoforms: TrxR1, located in the cytosol (55 kDa), the mitochondrial isoform TrxR2 (56.2 kDa), and TrxR3 (65 kDa), which is localized in the nucleus. TrxR2 contains an additional N-terminal sequence responsible for mitochondrial translocation. TrxR3 can reduce both oxidized Trx and oxidized glutathione (GSSSG), and is thus often referred to as thioredoxin-glutathione reductase [36,37,38,39]. TrxR3 contains an additional glutaredoxin domain at the N-terminus. This allows for the reduction of mixed disulfides in proteins formed by glutathionylation [38,39].

### 2.3. Functions of the Thioredoxin System

The Trx system reverses protein oxidations and can scavenge ROS (Figure 1), thus maintaining cellular redox equilibrium and modulating oxidative signals. Oxidative signals are integral to many signal transduction cascades, with H_2_O_2_ playing a key role due to its relatively long half-life, its ability to diffuse through membranes, and its primary attack on free thiols, leading to disulfide bond formation [4]. The reversibility of such oxidations is crucial for signaling, with Trx being pivotal in reducing disulfide bonds, thereby enabling the switching on and off of these signals.

#### 2.3.1. Regulation of Thioredoxin Expression and Activity

To rapidly and precisely adapt to changes in redox equilibrium, Trx activity can be regulated at various levels, including gene expression, posttranslational modifications, and protein–protein interactions [23].

The transcription factors nuclear factor erythroid 2-related factor 2 (Nrf2), TATA-binding protein (TBP), and cAMP response element-binding protein (CREB) can regulate the Trx system at the level of gene expression [29,40,41]. These transcription factors are activated by cellular stress, such as the accumulation of ROS. Upon activation, they bind to the antioxidant response element (ARE) in the promoter region of Trx and initiate transcription. There are also feedback mechanisms between these transcription factors and Trx. Active reduced Trx promotes Nrf2 binding by reducing cysteine residues in the DNA-binding domain of small musculoaponeurotic fibrosarcoma proteins (sMAF). The sMAF proteins can form a heterodimer with Nrf2 and stabilize its DNA binding, further enhancing Trx transcription [42]. Expression of Nrf2 and subsequently Trx can also upregulated by the aryl hydrocarbon receptor (AhR). AhR is a ligand-activated transcription factor belonging to the Per-ARNT-Sim (PAS) family. In its inactive state, AhR is part of a complex with heat shock protein 90 (Hsp90), the aryl hydrocarbon receptor interacting protein (AIP), and p23 in the cytosol. Upon ligand binding, which includes a range of polycyclic hydrocarbons such as benzo[a]pyrene, dioxins, and furans, as well as indole derivatives, AhR is released and translocates to the nucleus. In the nucleus, AhR forms a dimer with the aryl hydrocarbon receptor nuclear translocator protein (ARNT) and can induce anti-stress responses. Among other functions, AhR can induce the expression of Nrf2 and the Nrf2 target Trx [43,44].

In addition to its redox-active cysteine residues (Cys32 and Cys35), Trx has three non-active cysteine residues (Cys62, Cys69, and Cys73) that can be post-translationally modified, altering its activity. Trx can be glutathionylated at Cys73, preventing dimerization and inhibiting its enzymatic activity [45]. S-nitrosylation of Trx at Cys62, Cys69, or Cys73 also restricts its activity [46,47,48]. Trx can also be phosphorylated at threonine 100 (T100), influencing its activity [49].

Trx can also be inhibited through protein–protein interactions. Thioredoxin-interacting protein (Txnip) binds to the active site of Trx1 via its cysteine residue Cys247. This binding results in inactivation of Trx1 activity [29,50,51,52,53]. Txnip-mediated inhibition of Trx is involved in inflammation and aging [54,55].

#### 2.3.2. Regulation of Gene Expression via the Thioredoxin System

The majority of Trx1 is located in the cytosol, with a subpopulation in the nucleus [56]. Thus, Trx1 can act on transcription factors in the nucleus.

Trx1 reduces the redox factor-1 (Ref-1). Subsequently, Ref-1 reduces a conserved cysteine in the DNA-binding domain of the transcription factor activator protein-1 (AP-1), enhancing its DNA-binding activity. This redox-sensitive mechanism is crucial for AP-1 activation and the induction of AP-1 target gene expression [57,58,59,60]. 

Trx1 and reduced Ref-1 play an essential role in stabilizing the hypoxia-inducible factor α (HIF-1α). Once stabilized, HIF-1α forms a heterodimer with HIF-1β. The HIF-1α/HIF-1β dimer binds to hypoxia response elements (HREs) and initiates the transcription of genes involved in cellular adaptation to hypoxia [61,62]. 

**Figure 3:**
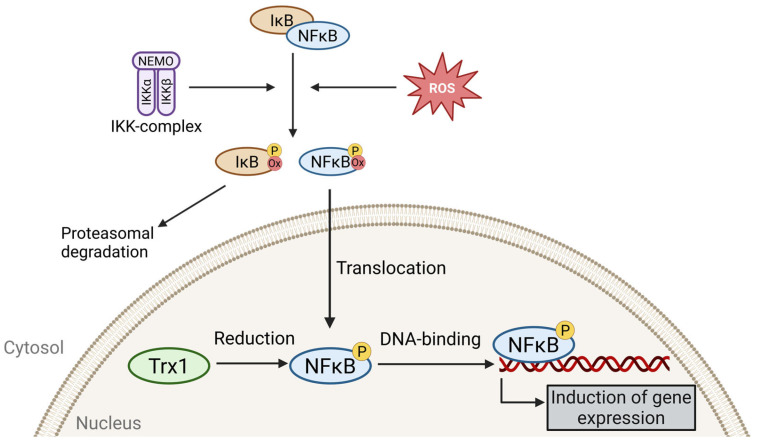
Phosphorylation of the inhibitor of NF-κB (IκB) by the IκB kinase (IKK) complex leads to its degradation. Oxidation of IκB can further enhance this process, resulting in the release of the nuclear factor kappa B (NF-κB). A pro-oxidative environment and oxidation of NF-κB promote its translocation to the nucleus. In the nucleus, NF-κB is reduced by thioredoxin 1 (Trx1) to ensure optimal DNA binding. P = phosphorylation; ox = oxidation. The figure was created with the assistance of BioRender.com.

In addition to AP-1 and HIF1α, nuclear factor kappa B (NF-κB) is a crucial redox-sensitive transcription factor. NF-κB refers to dimeric transcription factors within the Rel family [59]. In the absence of activating signals, NF-κB is sequestered in the cytoplasm by binding to the inhibitor of NF-κB (IκB). Upon activation of the IκB kinase (IKK) complex, IκB is phosphorylated, marked for ubiquitination, and subsequently degraded. Degradation of IκB results in the exposure of the nuclear localization sequence of NF-κB, facilitating its translocation into the nucleus [63,64]—a process enhanced by a pro-oxidative environment in the cytosol and the oxidation of thiol groups within NF-κB [3,65,66,67,68]. In the nucleus, NF-κB has to be reduced to bind optimally to DNA, a task performed by Trx1 (Figure 3) [3,26,59]. 

#### 2.3.3. Regulation of Cell Death via the Thioredoxin System

**Figure 4:**
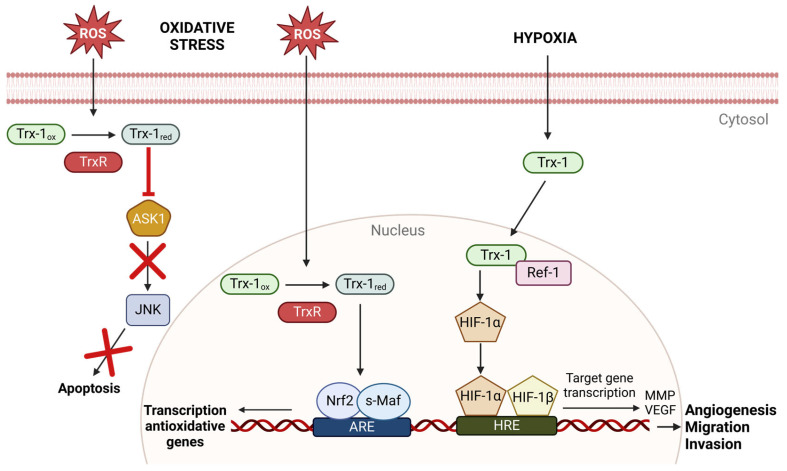
The thioredoxin (Trx) system modulates various signaling pathways in response to hypoxia and oxidative stress, leading to the inhibition of apoptosis and the induction of the anti-antioxidant defense. Additionally, it promotes angiogenesis, cell migration, and invasiveness, which can support tumor proliferation and metastasis. Trx1_red_ = reduced thioredoxin; Trx_ox_ = oxidized thioredoxin. The figure was created with the assistance of BioRender.com.

Trx influences the induction of apoptosis. In the cytosol, Trx1 can bind to the N-terminal non-catalytic region of apoptosis signal-regulating kinase 1 (ASK1), a mitogen-activated protein kinase kinase kinase (MAP3K). This binding inhibits ASK1, preventing it from activating the c-Jun N-terminal kinase (JNK) and the p38 MAP kinase pathway, which is necessary to initiate apoptosis. The interaction between Trx1 and ASK1 highly depends on the redox status of Trx1, with only the reduced form of Trx binding to ASK1 [29,69,70]. In addition, Trx1 can directly alter the stability of ASK1 by inducing the ubiquitination and degradation of ASK1. In this process, Trx1 interacts through a free thiol in its active site (Cys32 or Cys35) with a thiol group (Cys250) in ASK1 (Figure 4) [70,71].

Trx2 is crucial in controlling the accumulation of mitochondrial ROS. The absence of Trx2 leads to ROS accumulation, cytochrome c release from mitochondria, and induction of apoptosis [72]. Trx2 can also interact with ASK1 in mitochondria, blocking its activity. This process is similar to the inhibition of ASK1 in the cytosol by Trx1 [73]. Of note, Trx2 can maintain Bcl-x_L_ levels in a redox-independent manner, protecting the outer mitochondrial membrane from permeabilization and thereby preventing the induction of apoptosis [74].

### 2.4. Secretion of Thioredoxin

In response to increased oxidative stress, Trx can be secreted by cells, although the precise mechanisms are not yet fully understood [75,76,77,78,79,80]. Thus, serum or plasma levels of Trx serve as a reliable, minimally invasive marker for oxidative stress in various diseases [75,76,81]. Trx serum levels are elevated in patients with hepatocellular carcinoma (HCC) and pancreatic cancer [82,83]. Since these levels return to normal after tumor resection, the tumor itself is likely the main source of the secreted Trx [82]. Elevated levels of extracellular Trx have been also detected in patients with acquired immunodeficiency syndrome (AIDS). These Trx levels negatively correlate with reduced glutathione (GSH) levels, suggesting that HIV-infected individuals with AIDS experience elevated systemic oxidative stress [84,85]. Moreover, Trx is not only a marker in HIV infection but also plays a role in AIDS pathogenesis by reducing a disulfide bond in the HIV envelope glycoprotein gp120, facilitating CD4 binding and T cell entry [86,87]. In contrast to intracellular Trx, extracellular Trx does not appear to directly influence tumor proliferation. However, extracellular Trx provides protective effects against TNF- and ROS-induced apoptosis [88,89]. Extracellular Trx protects the tumor from the immune system [79,90]. Although most extracellular Trx is rapidly oxidized, it is likely that it can intercept therapeutic agents before they enter cells, potentially affecting the patient’s response to treatment.

### 2.5. The Thioredoxin System in Cancer

The Trx system is undoubtedly a double-edged sword. On one hand, it protects against oxidative damage, preventing the oxidation of macromolecules such as proteins, lipids, and nucleic acids. This protects cells from the accumulation of mutations, thereby defending the organism from the development of malignancies. 

An existing malignoma benefits from these protective mechanisms. Tumors often exhibit elevated ROS levels due to their metabolism. Trx prevents this increased ROS production from damaging and killing tumor cells. This increased proliferation due to the activation of transcription factors like NF-κB or AP-1 leads to faster tumor growth. Additionally, HIF-1α significantly promotes angiogenesis and metastasis formation. Activated HIF-1α induces various target genes such as vascular *endothelial growth factor* (*VEGF*) and *matrix metalloproteinases* (*MMPs*) genes. VEGF promotes angiogenesis, while MMPs facilitate invasion and metastasis [91,92,93,94,95]. 

There is a correlation between the overexpression of components of the Trx system and multidrug resistance (MDR) [96]. In pancreatic cancer cells, higher Trx expression protects against cisplatin-induced apoptosis [97,98,99]. In *KRAS* wild-type colorectal carcinoma cells, increased TrxR2 expression is involved in the development of MDR [100]. It has been shown that Trx expression is increased to protect various cancers, leading to increased tumor aggressiveness and poorer overall survival [62,101,102,103], including in colorectal carcinoma (CRC) [104,105,106], lung cancer [102,107], pancreatic cancer [83], gastric cancer [108,109,110,111], hepatic cancer [82,112], breast cancer [113,114,115], and tumors of the hematopoietic system [26,116,117,118,119]. A correlation between the thioredoxin system and resistance to paclitaxel has also been observed. In ovarian cancer cells, it has been shown that Trx1 binds to the transcription factor forkhead box protein O1 (FOXO1) and induces its translocation to the nucleus, and thereby promotes the expression of FOXO1-dependent genes that protect the tumor from cell death [120]. Trx can also protect breast cancer cells from tamoxifen-induced apoptosis by directly scavenging H_2_O_2_ via Prx or inducing estrogen-dependent and estrogen-independent redox-sensitive survival pathways [121]. Increased expression of the Trx system proteins is also associated with resistance to doxorubicin, docetaxel, and tamoxifen [62,99,122,123]. 

Therefore, the overexpression of Trx system components not only results in more aggressive, rapidly growing, and highly metastatic tumors, but also significantly limits treatment options and reduces patient survival. Thus, the individual components of the Trx system represent viable targets for cancer therapy.

## 3. Manipulation of the Thioredoxin System—Potential Therapeutic Approaches

The Trx system is essential for maintaining cellular redox balance and, together with Prx, acts as a crucial ROS scavenger. On one hand, it helps control oxidative signaling pathways, prevent oxidative damage, and reduce mutation accumulation, thus ensuring normal cell function and preventing cancer. On the other hand, the Trx system can protect established tumors from cell death and even promote their proliferation. Cancer cells often exhibit higher ROS levels, and the Trx system protects these cells from oxidation-induced cell death. Additionally, since the Trx system regulates proliferation-promoting transcription factors, it can further enhance the proliferation of malignancies.

### 3.1. Inhibition of Thioredoxin

Inhibiting Trx presents a promising target. Particularly interesting are the cysteines in the active site (Cys32 and Cys35), as well as cysteines Cys62, Cys69, and Cys73, which, although not located in the active site, regulate Trx activity.

#### 3.1.1. 1-Methyl Propyl 2-Imidazolyl Disulfide (PX-12)

**Figure 5:**
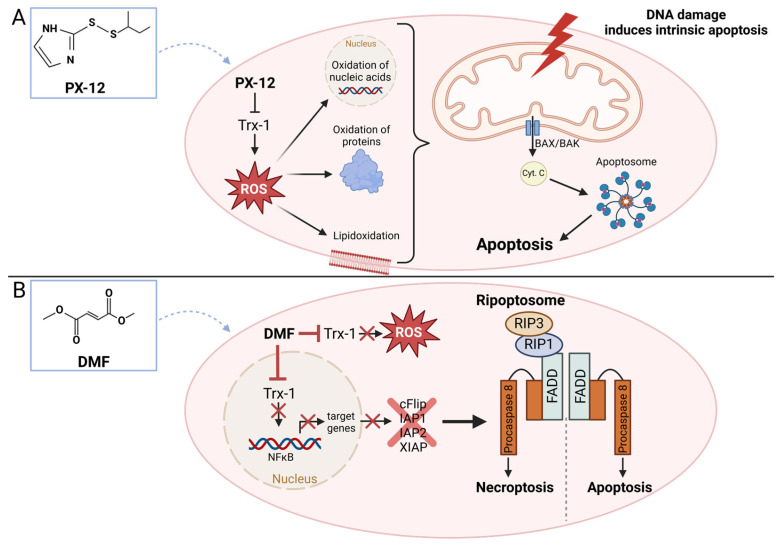
Schematic representation of the mechanism of action of PX-12 and dimethyl fumarate (DMF). (**A**) PX-12 inhibits thioredoxin 1 (Trx1). This leads to the accumulation of reactive oxygen species (ROS), which oxidizes macromolecules such as lipids, proteins, and nucleic acids. These oxidative damages (particularly the accumulation of DNA damage) induce the intrinsic apoptosis cascade. The pro-apoptotic Bcl-2 family members BAX (Bcl-2-associated X protein) and BAK (Bcl-2 homologous antagonist/killer) oligomerize and form a pore in the outer mitochondrial membrane, leading to a collapse of the membrane potential and the release of cytochrome C (Cyt C). This release of Cyt C induces the formation of the death platform called the apoptosome and the induction of cell death. (**B**) DMF also inhibits Trx1. As a result, the redox-sensitive transcription factor NF-κB can no longer be reduced in the nucleus. NF-κB can then no longer effectively bind to its binding sites in the promoter regions of its target genes. This leads to a decreased expression of the anti-apoptotic proteins cFLIP (FLICE-like inhibitory protein) and the inhibitor of apoptosis (IAP) proteins IAP1, IAP2, and XIAP. The absence of IAPs allows the formation of the death platform called the ripoptosome. This can induce both apoptosis and necroptosis through the receptor-interacting serine/threonine-protein kinases RIP1 and RIP3, if the apoptosis pathway is blocked. The figure was created with the assistance of BioRender.com.

PX-12 is a small molecule originally discovered through a screening evaluating disulfide compounds for their inhibition of cancer cell proliferation [124,125,126]. PX-12 binds to Cys73 of Trx1, inhibiting the reduction of oxidized Trx1 by TrxR1. This leads to the accumulation of inactive, oxidized Trx1 [127,128]. It has been shown that PX-12 sensitizes acute myeloid leukemia (AML) cells to arsenic trioxide-induced apoptosis [129]. In addition, PX-12 alone can induce cell death in AML cells [129]. In acute lymphoblastic leukemia (ALL) cells, PX-12 was also shown to induce cell death [130]. In an osteosarcoma model, PX-12 was also able to induce cell death and prevent metastasis formation [131]. In all these models, it is hypothesized that inhibition of Trx1 leads to a significant accumulation of ROS, which induces mitochondria-mediated apoptosis (Figure 5A). 

The efficacy and maximum tolerated dose of PX-12 in patients with advanced or metastatic cancer were investigated in a Phase I clinical study (NCT00736372) (Table 1) [132]. However, a Phase II clinical study in patients with advanced pancreatic cancer was terminated early due to low Trx1 expression in the patients and a lack of treatment efficacy with PX-12 (NCT00177242) (Table 1) [133]. The authors of the study suggest selecting patients with elevated Trx1 expression for future studies and then repeating the trial. However, no data are currently available on this. It remains to be seen whether PX-12 will find a clinical application in cancer patients.

#### 3.1.2. Dimethyl Fumarate (DMF)

Treatment of cells with DMF leads to modification of Cys73 in Trx1, similar to treatment with PX-12. DMF causes monomethyl-succinylation of this cysteine, resulting in the inactivation of Trx1. Active Trx1 is required in the nucleus to reduce NF-κB, enabling optimal binding to its DNA binding sites [26]. T-cell lymphomas with constitutive NF-κB activation, such as T-ALL and cutaneous T-cell lymphoma (CTCL), show dependence on NF-κB. When NF-κB is inhibited, these cells undergo cell death [8,26,134]. Inhibition of NF-κB by DMF leads to decreased expression of various target genes, including gens of anti-apoptotic proteins such as inhibitors of apoptosis (*BIRC2* encoding for IAP1, *BIRC3* encoding for IAP2, and *X-Linked Inhibitor Of Apoptosis* encoding for XIAP) and the gene for the FLICE-like inhibitory protein (*CFLAR* encoding for cFLIP). The downregulation of these proteins leads to the assembly of an intracellular death platform, the ripoptosome. The ripoptosome, whose activity is regulated by cFLIP, subsequently induces apoptosis or necroptosis if the apoptotic pathway is inhibited (Figure 5B) [26]. These data were also confirmed in a CTCL mouse model. The primary tumor showed significantly slower growth. In addition, metastasis was shown to be almost completely blocked [134].

A multicenter Phase II clinical study (NCT02546440) also demonstrated the efficacy of DMF in patients with CTCL (Table 1). DMF demonstrated its efficacy, particularly in patients suffering from the highly aggressive form of CTCL known as Sézary syndrome. Prolonged survival was observed in these patients. It is important to note that these patients also exhibited high constitutive NF-κB activation. The quality of life of the patients significantly improved, as skin lesions rapidly regressed immediately after the initiation of treatment. In addition, DMF is administered orally, which significantly reduces the burden on patients. Moreover, DMF showed almost no undesirable side effects in these patients [135]. A combination with Bcl-2 inhibitors was also investigated. It was shown that DMF and the Bcl-2 inhibitor ABT-199 (Venetoclax), as well as a combination with ABT-263 (Navitoclax), synergistically induced cell death in malignant T-cells isolated from CTCL patients [136]. DMF and ABT-199 also synergistically induced cell death in a mouse model [136], suggesting that this combination should be further investigated in clinical studies, as both DMF and ABT-199 are already in clinical use.

**Table 1:** Clinical studies on the inhibition of thioredoxin (Trx) for cancer therapy (for details, see the sections above).

Study Title	Study Number	Status	Published Results
A Trial of PX-12 in Patients With a Histologically or Cytologically Confirmed Diagnosis of Advanced or Metastatic Cancer	NCT00736372	Completed	[132]
Study of Gefitinib and Docetaxel as Salvage Therapy in Advanced Pancreatic Carcinoma	NCT00177242	Prematurely terminated	[133]
Study on Therapy With Dimethylfumarate (DMF) in Patients With Cutaneous T Cell Lymphoma (CTCL) (DMF-CTCL)	NCT02546440	Completed	[135]

#### 3.1.3. 4-(benzothiazol-2-yl)-4-hydroxycyclohexa-2,5-dienone (PMX464)

PMX464 (previously named AW464) is a benzothiazole-substituted quinoline compound capable of binding all five cysteines in Trx-1, including Cys32 and Cys35 in the active site [137,138,139]. Consequently, PMX464 is a potent Trx-1 inhibitor currently undergoing preclinical evaluation [139]. Studies on colorectal and breast cancer cell lines have shown that PMX464 induces cytotoxic and anti-proliferative effects [140,141]. In colon carcinoma cells, it has been shown that the effect of PMX464 is limited under normoxic conditions but becomes cytotoxic under hypoxia. Additionally, fibroblasts (even under hypoxic conditions) are relatively resistant to PMX464 compared to the tumor cell lines [141]. However, the identification of an optimal dose of the inhibitor will therefore be crucial to effectively use it as a hypoxic anti-tumorogenic therapy.

#### 3.1.4. Suberoylanilide Hydroxamic Acid (SAHA)

Histone deacetylase inhibitors (HDACis) like SAHA are agents that modify chromatin. Gene expression regulation depends on histone acetylation and deacetylation, mediating interactions between histone complexes and chromatin. HDACis inhibit histone deacetylation, leading to modulation of gene expression and prevention of chromatin condensation [128,142,143,144]. SAHA is a polar compound from the hydroxamate group of HDACis [145]. In prostate cancer cells, it has been shown that SAHA induces the expression of the Trx inhibitor *TXNIP*. TXNIP, in turn, inhibits Trx-1 and leads to oxidative stress in the tumor cells [146,147]. Therefore, indirect inhibition of Trx-1 via SAHA is an interesting option for tumor treatment.

#### 3.1.5. Sodium Butyrate

Sodium butyrate is a short-chain fatty acid mainly produced by bacteria. It has been shown to reduce the activity of Trx-1 in colon carcinoma cells, while the activity of Trx-1 in healthy mucosal cells did not change after sodium butyrate treatment [148,149]. Sodium butyrate inhibits HDAC activity, acting as an HDAC inhibitor, leading to histone hyperacetylation. Similar to SAHA, sodium butyrate induces the expression of the natural Trx inhibitor TXNIP, which restricts Trx activity [149]. These results were also confirmed in in vivo studies, where treatment with sodium butyrate led to a reduction in tumor growth [104,148]. 

#### 3.1.6. Isoforretin A (Iso A)

Diterpenoids are terpenoid compounds consisting of 20 carbon atoms arranged in four isoprene units. Iso A is a diterpenoid compound isolated from *Isodon forrestii* var. *forrestii*, a medicinal herb from China. Iso A can inhibit Trx-1, leading to an increase in ROS levels within cells, resulting in oxidative stress and cell death. In a colon carcinoma model, it was shown that Iso A not only inhibits Trx-1 activity but also induces chromatin condensation and fragmentation, leading to cell death [150].

#### 3.1.7. Diallyl Trisulfide

Diallyl trisulfide, also known as allitridin, is an organosulfur compound derived from garlic. It can be formed through the hydrolysis of allicin. Through Michael addition, diallyl trisulfide directly conjugates to the Cys32 and Cys35 residues in the active site of Trx-1, thereby inhibiting it [151].

The predominant cause of high mortality in triple-negative breast cancer (TNBC) is metastasis. It is suggested that Trx-1 plays an important role in breast cancer metastasis. Analyses in a nude mouse model of spontaneous and induced breast cancer metastasis showed that diallyl trisulfide leads to inhibition of Trx-1 and suppresses metastasis [151]. In addition, it has been demonstrated that diallyl trisulfide sensitizes glioblastoma cells to radiotherapy. This is likely directly related to the inactivation of Trx-1 and a weakening of the oxidative defense by diallyl trisulfide [152].

#### 3.1.8. Eprenetapopt (APR-246, PRIMA-1Met)

In addition to thioredoxins, glutaredoxins (Grx) are involved in reducing disulfide bonds and reversing protein oxidations. There are four human Grx proteins: Grx1, located in the cytosol, mitochondrial intermembrane space, and nucleus; Grx2, located in the cytosol, mitochondria, and nucleus; Grx3, located in the cytosol and nucleus; and Grx5, located in the cytosol and mitochondria [153]. Grx contains a disulfide bridge in its active site and uses glutathione as a cofactor. Unlike Trx, Grx is reduced by glutathione rather than specific reductases. Due to the similar functions of Trx and Grx, and since Grx can serve as a backup system for TrxR to restore Trx activity, inhibitors that block both Trx and Grx are of particular clinical interest [153]. Eprenetapopt (APR-246, PRIMA-1Met), a small molecule originally developed to restore the activity of mutated, inactive p53, also inhibits Trx1 and Grx1 [154]. The extent of its effectivity on tumors in relation to Trx/Grx inhibition requires further investigation. Other interesting Grx inhibitors include the fungal toxin sporidesmin [155] and the chloroacetamido compound CWR-J02 [156].

Thus, the inhibition of Trx and Grx are potential targets for cancer treatment. PX-12 and DMF are two promising candidates for inhibiting Trx that are currently in clinical trials. However, it must always be considered that Trx and Grx play important physiological roles, which will undoubtedly be affected by the application of an inhibitor. Therefore, it is crucial to determine the correct dosage and achieve the highest possible specificity towards the tumor

### 3.2. Inhibition of Thioredoxin Reductase

TrxR is upregulated in many tumors, attracting increasing interest as an anti-cancer target. TrxR has an N-terminal redox center (Cys59/Cys64) that is sterically inaccessible and unsuitable for inhibitor development. Mammalian TrxRs possess an essential selenocysteine (Sec) residue at the C-terminus (Cys497/Sec498), which forms another redox-active site with an adjacent cysteine (Cys) via a selenolthiol/selenenylsulfide exchange reaction. This structure is on the protein surface, making it more accessible and suitable for inhibitor development [15].

#### 3.2.1. Ethaselen

Ethaselen is an organoselenium TrxR inhibitor that exerts its antitumor effects through the induction of intrinsic (mitochondrial-induced) apoptosis in several cancer cells, including lung, stomach, cervical, and prostate cancer [157,158]. Ethaselen is currently being investigated in a Phase I clinical trial for advanced non-small cell lung cancer with elevated thioredoxin reductase activity (NCT02166242) (Table 2). The focus of this trial is on progression-free survival, quality of life, overall survival, and drug safety. The recruitment phase has been completed, but no results have been published yet.

#### 3.2.2. Metal Complexes Targeting Thioredoxin Reductase—Auranofin

Various metal complexes that influence TrxR activity are used in cancer therapy, with platinum and gold complexes being particularly significant. Gold complexes, formed from gold ions with specific electronic properties, interact with reduced macromolecules, including reduced TrxR, and inhibit their function. Among these, the gold compound auranofin is the most effective TrxR inhibitor [15,39,159,160,161]. Platinum complexes (cisplatin, carboplatin) irreversibly inhibit TrxR. Interestingly, the activity of other reductases such as glutaredoxin or glutathione (GSH) reductase is not inhibited. This is due to platinum complexes having a higher affinity for the selenol residue in TrxR compared to the thiol groups present in other reductases [39,162]. These metal complex inhibitors efficiently block TrxR in both the cytosol and mitochondria. This blockade results in the release of ROS, which subsequently damages macromolecules such as lipids, proteins, and nucleic acids. Particularly, the inhibition of TrxR2 in the mitochondria leads to massive ROS production and mitochondrial damage. The electron transport chain (ETC) complexes are also affected, resulting in further increased ROS production and significantly impairing energy production via the ETC. 

**Figure 6:**
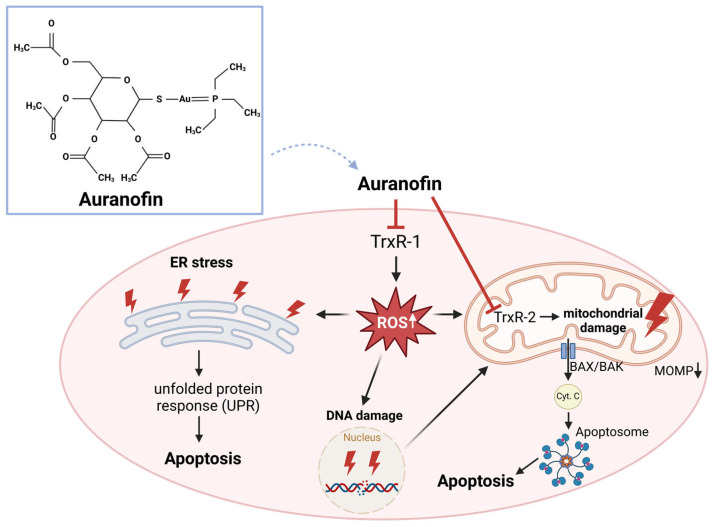
Auranofin inhibits thioredoxin reductase 1 (TrxR1), leading to reactive oxygen species (ROS) accumulation. Elevated ROS levels cause the accumulation of misfolded proteins in the endoplasmic reticulum (ER), triggering ER stress and the induction of the unfolded protein response (UPR), which can induce cell death. ROS also causes DNA damage, activating the intrinsic apoptotic pathway. This results in mitochondrial outer membrane permeabilization (MOMP) and the release of cytochrome C (Cyt C) from the mitochondrial intermembrane space through a pore formed by the Bcl-2-associated X protein (BAX) and the Bcl-2 homologous antagonist/killer (BAK), subsequently leading to the formation of the apoptosome and the induction of apoptosis. Auranofin further exacerbates this by inhibiting mitochondrial thioredoxin reductase 2 TrxR2, causing mitochondrial damage, including to the electron transport chain, which increases ROS production and oxidative damage.

For example, it has been shown that auranofin leads to an accumulation of ROS in colorectal carcinoma cells (Figure 6). This can sensitize malignant cells to radiation [163] and induce cell death by causing ROS-induced DNA damage [164]. Furthermore, it has been shown that auranofin can inhibit cancer cell proliferation, invasiveness, and metastasis, which has also been confirmed in colon carcinoma mouse models [163,165]. Also interesting are data from colon organoids. It was shown that cancer organoids responded much more effectively to auranofin compared to organoids derived from healthy tissue. Therefore, auranofin appears to exhibit a certain degree of tumor specificity [166]. In lung carcinoma cells, auranofin induces the accumulation of ROS and inhibition of glycolysis, leading to induction of cell death [167]. Auranofin also shows sensitizing effects in combination with other agents. In B-cells, high doses of L-ascorbate lead to autoxidation and the production of H_2_O_2_. This mechanism is mitigated by the Trx system. However, when TrxR is inhibited by auranofin, there is a significant induction of oxidative stress. Interestingly, in B-cells treated with the combination of auranofin and L-ascorbate, H_2_O_2_ reacts with iron (Fe)^2+^ in the so-called Fenton reaction, generating ^●^OH, which cause lipid peroxidation and subsequently trigger ferroptosis [168,169]. In ovarian cancer cells that were resistant to platinum treatment, ROS-dependent cell death could be effectively induced with auranofin in combination with selenite or tellurite [170]. In breast cancer cell lines, a synergistic effect of auranofin and mesupron, a small molecule that inhibits urokinase-type plasminogen activator (uPA), was demonstrated [171]. Due to promising results, four clinical trials with auranofin are currently underway for the treatment of chronic lymphocytic leukemia (NCT01419691), ovarian cancer (NCT01747798, NCT03456700), and recurrent lung cancer (NCT01737502). However, no results of the studies have been released yet (Table 2).

In the search for more efficient hydrophilic, water-soluble metal complexes, Casini and colleagues demonstrated that various mono- and binuclear gold(I) complexes with sulfonate and hydroxyl groups are capable of inhibiting TrxR [172,173] and inducing apoptosis in various cancer models [172,173,174,175,176].

#### 3.2.3. Curcumin

Curcumin is extracted from Curcuma longa, a plant species belonging to the ginger family (Zingiberaceae). anti-inflammatory and anticancer properties are attributed to curcumin. It has been reported that curcumin inhibits, among others, NF-κB and TrxR [177]. Curcumin hinders the activity of TrxR by binding to its active site and disrupting the electron transfer from NADPH to TrxR [178]. Several studies show that curcumin leads to an accumulation of ROS in tumor cells, inhibiting metastasis formation and inducing cell death and/or sensitizing the cells to radiation [179]. 

Curcumin exhibits limitations for potential clinical applications due to its poor water solubility. Therefore, analogues have been developed, such as WZ26, which inhibits TrxR and restricts the proliferation and survival of tumor cells [180]. Currently, additional analogues are being developed and tested [113].

Several clinical studies have already been conducted on curcumin. In a Phase I trial with theracurmin, a curcumin derivative that demonstrates higher bioavailability than curcumin, it was shown that a combination with irinotecan is safe and well-tolerated in patients with advanced solid tumors (NCT04028739) [181]. Another clinical study on patients with colorectal carcinoma and unresectable metastases demonstrated that a combination treatment of bevacizumab/FOLFIRI with a ginsenoside-modified nanostructured lipid carrier containing curcumin resulted in an overall 1-year survival rate of 87.4% (NCT02439385) [182]. In a Phase II clinical study, patients were treated either with FOLFOX (folinic acid/5-fluorouracil/oxaliplatin) or with a combination of FOLFOX and curcumin. The combination therapy showed no significant side effects and was deemed safe. Although a slightly improved overall survival rate was observed, there were no significant improvements in quality of life or reduction in neurotoxicity compared to the FOLFOX treatment alone (NCT01490996) [183] (Table 2). In additional studies that have been completed but have not yet released results, curcumin has been investigated for the treatment of colorectal carcinoma (NCT00027495, NCT01859858), prostate cancer (NCT03211104), and breast cancer (NCT03847623) (Table 2). Another study on the treatment of leukemia (NCT05045443) has completed the recruitment phase, while studies on cervical cancer (NCT06080841) and prostate cancer (NCT02064673) are still in the recruitment phase. Initial results are expected in 2025 and 2026, respectively (Table 2).

**Table 2:** Clinical studies on the inhibition of thioredoxin reductase (TrxR) for cancer therapy (for details, see the sections above).

Study Title	Study Number	Status	Published Results
Ethaselen for the Treatment of Thioredoxin Reductase High Expression Advanced Non-Small Cell Lung Cancers	NCT02166242	Recruitment completed; analysis pending; last update 2022	No data posted
Phase I and II Study of Auranofin in Chronic Lymphocytic Leukemia (CLL)	NCT01419691	Completed	No data posted
Auranofin in Treating Patients With Recurrent Epithelial Ovarian, Primary Peritoneal, or Fallopian Tube Cancer	NCT01747798	Completed	No data posted
Auranofin and Sirolimus in Treating Participants With Ovarian Cancer	NCT03456700	Recruitment completed; analysis pending; last update 2024	No data posted
Sirolimus and Auranofin in Treating Patients With Advanced or Recurrent Non-Small Cell Lung Cancer or Small Cell Lung Cancer	NCT01737502	Recruitment completed; analysis pending; last update 2023	No data posted
Theracurmin vs. Curcumin Bioavailability Study	NCT04028739	Completed	[181]
Avastin/FOLFIRI in Combination With Curcumin in Colorectal Cancer Patients With Unresectable Metastasis	NCT02439385	Completed	[182]
Combining Curcumin With FOLFOX Chemotherapy in Patients With Inoperable Colorectal Cancer (CUFOX)	NCT01490996	Completed	[183]
Curcumin for the Prevention of Colon Cancer	NCT00027495	Completed	No data posted
Effect of Curcumin on Dose Limiting Toxicity and Pharmacokinetics of Irinotecan in Patients With Solid Tumors	NCT01859858	Recruitment completed; analysis pending; last update 2023	No data posted
Comparison of Duration of Treatment Interruption With or Without Curcumin During the Off Treatment Periods in Patients With Prostate Cancer Undergoing Intermittent Androgen Deprivation Therapy	NCT03211104	Completed	No data posted
Effect of Preoperative Curcumin in Breast Cancer Patients (EPC)	NCT03847623	Unknown status	No data posted
Safety and Efficacy of Curcumin in Children With Acute Lymphoblastic Leukemia (CurcumPedALL)	NCT05045443	Recruitment completed; analysis pending	Results are expected in end of 2024
Curcumin Supplementation in Cervical Cancer	NCT06080841	Recruiting	Results are expected in 2025
Adjuvant Curcumin to Assess Recurrence-Free Survival in Patients Who Have Had a Radical Prostatectomy	NCT02064673	Recruiting	Results are expected in 2026

#### 3.2.4. Piperlongumine

Piperlongumine is an alkaloid extracted from long pepper plants (*Piper longum* L.). Piperlongumine inhibits TrxR [184]. It has been shown that piperlongumine-treated cancer cells accumulate ROS, leading to DNA damage, which sensitizes the cells to radiation [184,185]. These studies were conducted in vitro on cell lines. Only one study describes an additive effect with oxaliplatin in a colon cancer mouse model [186]. Due to the limited data, further studies are needed to determine if piperlongumine could be a viable option for tumor treatment and clinical application.

#### 3.2.5. Isodeoxyelephantopin

Isodeoxyelephantopin is a compound isolated from *Elephantopus scaber* L., a tropical flowering plant of the Asteraceae family. This medicinal plant is known for its anticancer properties [187]. Isodeoxyelephantopin inhibits TrxR and induces ROS accumulation, leading to the activation of the JNK signaling pathway and apoptosis. When combined with the glutamate-cysteine ligase inhibitor buthionine sulfoximine (BSO), which decreases GSH levels, the two substances synergistically induce apoptosis [188]. Additionally, treatment with isodeoxyelephantopin, both as a single agent and in combination with cisplatin, has reduced colorectal tumor growth in a mouse model [188].

## 4. Conclusions

Organisms have to manage ROS, which arise either as byproducts of enzymatic reactions or are intentionally generated for signaling. Maintaining redox balance is crucial to prevent oxidative damage and support effective signaling. The Trx system and other redox regulators maintain this balance. Dysregulation can lead to cellular damage and tissue degradation and promote malignancies. Tumor cells, with their altered metabolism, often have increased ROS production and express higher amounts of antioxidant proteins, including components of the Trx system. This makes the Trx system a potential target for cancer treatment. However, its essential role in healthy cells and in regulating oxidative signals related to survival and proliferation presents a challenge for therapeutic targeting.

Despite these challenges, Trx and TrxR inhibitors show promise in clinical trials (Table 1 and Table 2). Studies with PX-12 and DMF suggest that carefully selected doses can target malignant cells based on elevated Trx-1 expression to minimize the impact on healthy tissue. Combination therapies, like DMF with Bcl-2 inhibitors, further reduce the required therapeutic doses, helping to protect healthy cells. Indirect inhibition strategies, such as using HDACi SAHA to induce TXNIP, an endogenous Trx inhibitor, also offer the potential for selectively targeting tumors with elevated Trx levels. However, more research is needed to validate this approach. The same applies to TrxR; it is crucial to predominantly target tumor cells, as they are particularly reliant on the Trx system. Among TrxR inhibitors, metal complexes like auranofin stand out. Auranofin sensitizes tumor cells to oxidative stress, especially in combination with irradiation. Ongoing clinical trials are exploring its clinical utility. Similar considerations apply to the organoselenium TrxR inhibitor Ethaselen. The efficacy of curcumin is difficult to assess due to often inaccurate or misleading in vitro results. The mechanism of curcumin is complex, particularly the role of TrxR inhibition, as curcumin can influence many signaling pathways. However, clinical studies suggest curcumin could be a potential option for cancer therapy.

**Figure 7:**
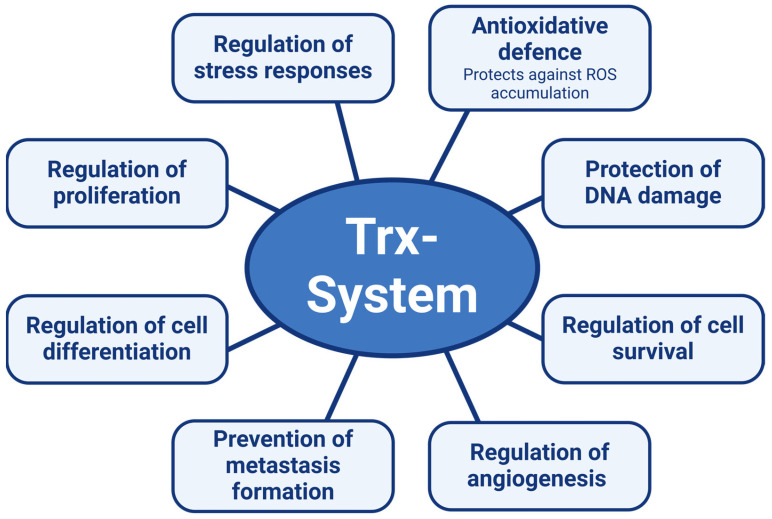
The thioredoxin (Trx) system regulates a plethora of signaling pathways. The figure was created with the assistance of BioRender.com.

In summary, the Trx system is vital for maintaining cellular redox balance, protecting against oxidative damage, and regulating cell survival, proliferation, and differentiation (Figure 7). It also plays a key role in neoangiogenesis, which is crucial for both organism survival and tumor growth. In tumors, the Trx system supports resistance to cell death, stress protection, and nutrient supply via neovascularization, increasing metastasis risk. This makes the Trx system a critical target for cancer therapy, although the challenge lies in specifically targeting the tumor’s Trx system or normalizing its upregulated activity.
